# Molecular and therapeutic advancements in Capicua (*CIC*)-rearranged sarcoma

**DOI:** 10.3389/fcell.2024.1416697

**Published:** 2024-05-31

**Authors:** Rovingaile Kriska M. Ponce, Cuyler Luck, Ross A. Okimoto

**Affiliations:** ^1^ Boston University Chobanian and Avedisian School of Medicine, Boston, MA, United States; ^2^ Department of Medicine, University of California, San Francisco, San Francisco, CA, United States; ^3^ Helen Diller Family Comprehensive Cancer Center, University of California, San Francisco, San Francisco, CA, United States

**Keywords:** CIC, CIC-DUX sarcomas, CIC-DUX4, CIC-NUTM1 sarcomas, CIC-rearranged sarcoma, Capicua (CIC)

## Abstract

Capicua (*CIC*)-rearranged sarcomas are an aggressive subset of undifferentiated round cell sarcomas. CIC::DUX4, the proto-typical CIC fusion oncoprotein is associated with rapid clinical progression and chemotherapy resistance leading to poor clinical outcomes. Recent studies have identified additional CIC fusions (CIC::NUTM1, CIC::FOXO4, and CIC::LEUTX) that largely retain CIC-binding specificity but leverage C-terminal binding partners (NUTM1, FOXO4, and LEUTX) to potentially activate transcriptional programs that drive oncogenesis. Moreover, the recent development of preclinical models to study CIC::DUX4 sarcoma have advanced our understanding of the underlying biological mechanisms and uncovered key dependencies that can be translated into rational therapies. In this review, we will highlight these recent advancements in *CIC*-rearranged sarcoma biology with a vision for clinical translation to improve patient outcomes.

## Introduction

Capicua (*CIC*)-rearranged sarcomas represent a rare and highly aggressive subset of undifferentiated small round cell sarcomas. The most frequently observed CIC-rearranged protein involves a fusion between the transcriptional repressor, *CIC*, and the double homeobox 4 gene, *DUX4*. Subsequent to their initial description, the World Health Organization (WHO) classified CIC::DUX4 sarcoma (CDS) as a “Ewing sarcoma-like” tumor based primarily on their morphological similarities to Ewing sarcoma (ES). Consequently, the clinical management of CDS often mimics ES, albeit with worse clinical outcomes relative to the latter. Since this initial classification, emerging data now indicates that CIC::DUX4 drives sarcomagenesis through a distinct transcriptional program compared to ES and other small round cell sarcomas. Moreover, there are divergent clinical manifestations and outcomes associated with CDS patients that support the notion that CDS is a distinct disease entity that warrants a well-defined clinical and molecular distinction from other small round cell sarcomas.

Here, we will review the molecular characteristics of CIC-rearranged proteins including CIC::DUX4, CIC::NUTM1, CIC::FOXO4, and CIC::LEUTX with a focus on fusion structure, function, and transcriptional targets. Moreover, we will report on the preclinical models that have been developed to better understand CIC fusion oncoproteins in the context of cancer and how these insights are fueling treatment strategies. Thereafter, we aim to compare and contrast the clinical manifestations between CIC fusions with an emphasis on the anatomical distribution and clinical outcomes among patients. These molecular and clinical observations will potentially provide a framework to develop consensus therapies for this underserved subset of human sarcoma.

## Molecular characteristics of CIC fusions

### Mechanisms of transcriptional control in *CIC*-rearranged sarcoma

Native wild-type (WT) Capicua (*CIC*) was initially discovered as a high-mobility-group (HMG) box transcriptional repressor in *Drosophila melanogaster*, where it operates as a MAPK-ERK signaling sensor to coordinate embryonic pattern formation (Jiménez et al., 2000). The mechanism by which *CIC* confers transcriptional repression in mammals has recently emerged through mass spectrometry (MS) analysis. Specifically, MS has delineated physical interactions with the SIN3-HDAC corepressor complex at CIC transcriptional target sites (Weissmann et al., 2018; Hwang et al., 2020). These studies have also suggested a mechanistic link between the CIC-SIN3/HDAC complex and SWI/SNF recruitment to *CIC* DNA foci (Hwang et al., 2020; Takemon et al., 2023). These early studies collectively suggest that CIC cooperates with SIN3/HDAC and the mSWI/SNF repressor complexes to transcriptionally silence target genes in mammalian systems. Upon recruitment and repressor complex assembly at CIC DNA binding sites, CIC silences highly conserved MAPK-ERK target genes through the recognition of TGAATGAA-like DNA-motifs (Forés et al., 2017). Upon MAPK activation, CIC is functionally suppressed through ERK–mediated degradation leading to the derepression of MAPK-ERK responsive genes. The underlying mechanism of CIC DNA binding and sequence recognition is thought to be regulated by its conserved HMG box and C1 domains, which enables high sequence specificity (Ajuria et al., 2011; Forés et al., 2017; Webb et al., 2022). The most well studied CIC targets include the *PEA3* family of transcription factors, namely *ETV1*, *ETV4*, and *ETV5*. In the context of cancer, genetic loss or functional suppression of CIC derepresses *ETV1*, *ETV4*, and/or *ETV5* in multiple subsets of human cancer such as lung, gastric, breast, and colorectal cancer, resulting in enhanced tumor proliferation, self-renewal, and invasion/metastasis (Okimoto et al., 2017; Lee et al., 2020; Yoe et al., 2020).

**FIGURE 1:**
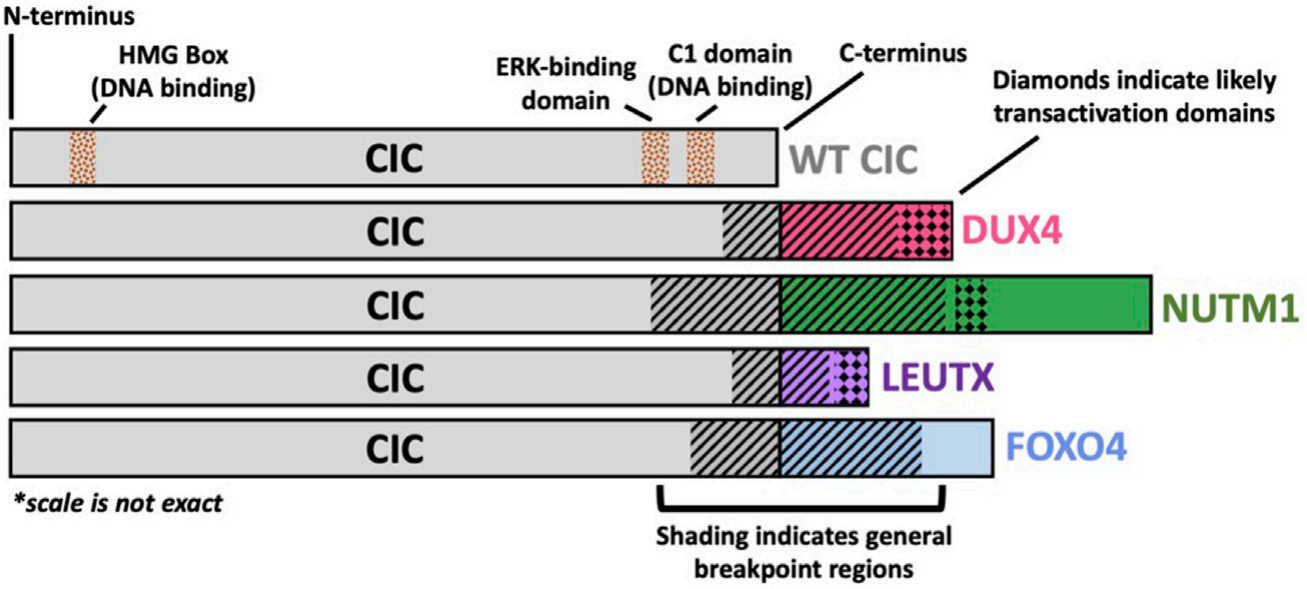
Structural Schematic of CIC Fusion Oncoproteins. Structural schematic of CIC fusions highlighting the highly conserved wild-type (WT) CIC region with variability in the fusion break point and c-terminal binding partner. The CIC::DUX4 fusion oncoprotein has been shown to retain WT CIC DNA binding specificity through its HMG box and C1 domain but acquires transcriptional activating capacity via DUX4-mediated p300 recruitment to CIC binding sites. The C-terminal bindings partners, NUTM1, LEUTX, and FOXO4 have previously been reported to interact with p300/CBP.

Despite its predominant function as a transcriptional repressor, the role of *CIC* dysregulation in cancer was first described through its activating capacity as a fusion oncoprotein in 2006 (Kawamura-Saito et al., 2006). Specifically, using *in vitro* and *in vivo* transformation models, Takuro Nakamura and colleagues functionally characterized the first patient-derived *CIC* rearrangement, namely the CIC::DUX4 fusion oncoprotein. Structurally, the CIC::DUX4 fusion almost always retains >90% of native CIC, including the HMG box and C1 domain, and is conjoined with the C-terminal trans-activation domain of the double homeobox four gene DUX4 as a result of translocations t (4; 19) (q35; q13) or t (10; 19) (q26; q13) (Kawamura-Saito et al., 2006; Specht et al., 2014; Antonescu et al., 2017) (Figure 1). Through this fusion, the C-terminal end of CIC is replaced with the DUX4 transactivating domain that interacts with the p300/CBP complex. Thus, it is reasonable to hypothesize that these structural changes shift the interaction from the SIN3-HDAC and SWI/SNF repressor complexes to a p300/CBP dominant interaction, which results in transcriptional activation of CIC target genes. Thus, it is reasonable to conceive that CIC::DUX4 attains transcriptional activating capacity through a conserved DUX4 recruitment of p300/CBP, which induces histone H3 acetylation to enable target gene activation (Choi et al., 2016; Bosnakovski et al., 2021). Consistent with this, we and others have observed that global CIC::DUX4 binding overlaps with H3K27ac marks in patient derived CIC::DUX4 cell lines, again suggesting that p300/CBP may in part confer activating capacity (Thomas et al., 2023; Bakaric et al., 2024). Collectively, CIC co-opts the activating capacity of the p300/CBP associated transcription factor, DUX4, to upregulate key target genes including *PEA3* family members, cell cycle genes such as *CCND2* and *CCNE1,* and negative MAPK regulators including *DUSP4* and *DUSP6* that drive malignant phenotypes in aggressive undifferentiated round cell sarcomas (Kawamura-Saito et al., 2006; Yoshimoto et al., 2017; Okimoto et al., 2019; Ponce et al., 2022). Collectively, these structure-function studies reveal that CIC::DUX4 largely retains CIC DNA-binding specificity while transforming its native repressor function (SIN3-HDAC and SWI/SNF) into a potent oncogene in part through DUX4 mediated p300/CBP recruitment.

### Binding partners in CIC fusion oncoproteins

Since the initial discovery of CIC::DUX4, other CIC fusions have been reported including CIC::NUTM1, CIC::FOXO4, and CIC::LEUTX. With some variability in the 3’ *CIC* breakpoint, these CIC fusions largely retain the majority of WT *CIC*, including its HMG box domain, but replace the C-terminal end of CIC with components of another transcriptional regulator, including NUT midline carcinoma family member 1 (NUTM1), forkhead box O4 (FOXO4), or leucine twenty homeobox (LEUTX). Intriguingly, these *CIC* binding partners are well-defined developmentally regulated transcription factors that interact with p300/CBP (van der Heide and Smidt, 2005; Shiota et al., 2018; Gawriyski et al., 2023). Thus, it is reasonable to anticipate that these CIC-rearranged proteins can potentially operate in a similar fashion to CIC::DUX4 fusion oncoproteins.

CIC::NUTM1 was first reported in 2016 in a molecular analysis of primitive neuroectodermal tumors of the central nervous system (CNS), where two patients harbored a fusion between *CIC* exon 16 and *NUTM1* exon 4 (Sturm et al., 2016). Since then, CIC::NUTM1 fusions have been identified in at least an additional one CNS patient and twenty sarcoma patients, where the chimera occurs between *CIC* exons 16, 17, 18, and 20 and *NUTM1* exons 2, 3, 4, 5, and 6 (Mangray et al., 2018; Schaefer et al., 2018; Watson et al., 2018; Le Loarer et al., 2019; Biederman et al., 2022; Yang et al., 2022; Ma et al., 2023; Sievers et al., 2023) (Figure 1). While functional studies illuminating molecular pathology are limited, non-CIC::NUTM1 fusions have been implicated in NUTM1 midline carcinoma, specifically the BRD4::NUTM1 fusion, with p300 recruitment and subsequent histone acetylation to be a hypothesized mechanism of oncogenic activation (Reynoird et al., 2010; French, 2018). Structurally, CIC::NUTM1 fusions retain the CIC HMG box domain, but in contrast to CIC::DUX4, CIC::NUTM1 fusions appear to not universally harbor the CIC C1 domain, which has been previously linked to DNA-binding and target gene specificity (Forés et al., 2017). Interestingly, transcriptional profiling of patient-derived CIC::DUX4 and CIC::NUTM1 fusions demonstrated that these two groups shared some similarity but slightly diverged at the distal hierarchical branches (Watson et al., 2018). These findings suggest that while CIC::DUX4 and CIC::NUTM1 may share transcriptional targets, there may be key distinctions that molecularly differentiate these two fusion oncoproteins. Thus, a deeper and more comprehensive analysis may reveal key molecular distinctions that uniquely associate with each fusion. For example, if and/or how the C1 domain of CIC contributes to target gene specificity in the context of CIC fusions (CIC::DUX4 retains the C1 domain; CIC::NUTM1 does not retain the C1 domain). Additionally, this comparison could highlight key distinctions between how *CIC* binding partners, including *DUX4* and *NUTM1*, impact fusion specific target gene regulation. Future comparative studies aimed at addressing these questions is warranted as we believe that the molecular underpinnings of how these fusions mechanistically regulate cellular transformation may reveal key insights to better understand both the clinical features and therapeutic approaches for these patients.

The more rare CIC::FOXO4 fusion has been reported in a total of four patients, with breakpoints identified in three patients occurring between *CIC* exons 19 or 20 and *FOXO4* exons 2 or 3 (Brohl et al., 2014; Sugita et al., 2014; Babkoff et al., 2023) (Figure 1). Thus, similar to CIC::DUX4 fusions, CIC::FOXO4 oncoproteins structurally share CIC DNA-binding elements (HMG-box and C1 domains), suggesting that CIC DNA-binding specificity and target gene regulation may be conserved relative to CIC::DUX4. While little is known about the molecular mechanisms of CIC::FOXO4 fusion oncoproteins, FOXO4 (also known as AFX) and FOXO1 (also known as FKHR) chimeras have been identified in MLL-rearranged acute leukemia (AFX::MLL) and alveolar rhabdomyosarcoma (PAX3::FOXO1) (Borkhardt et al., 1997; Sorensen et al., 2002). Similar to DUX4 and NUTM1, p300 has been shown to interact with the FOXO family of transcription factors (van der Heide and Smidt, 2005). Not only was FOXO4 depicted to co-immunoprecipitate with p300 in a peroxide-induced, redox-dependent manner, but it was also recently shown that a single cysteine in the activation domain of PAX3::FOXO1 is important for the recruitment of p300/CBP to chromatin, which was also conserved in other members of the FOXO family, including FOXO4 (Dansen et al., 2009; Asante et al., 2023). We fully anticipate that with improved CIC::FOXO4 models, we will better understand the molecular mechanisms of CIC::FOXO4 fusions and how they contribute to sarcomagenesis.

A fourth CIC fusion has been identified in sarcoma and non-sarcoma cancers—CIC::LEUTX. Published patient case reports have identified the CIC::LEUTX chimera to occur between *CIC* exon 20 and *LEUTX* exon 3 across the different cancers of renal sarcoma, spinal cord sarcoma, angiosarcoma, central nervous system embryonal tumors, and pediatric-type high-grade neuroepithelial tumors (Huang et al., 2016; Hu et al., 2020; Lake et al., 2020; Song et al., 2022; Sievers et al., 2023; Tang et al., 2023) (Figure 1). Interestingly, LEUTX is a developmentally regulated transcriptional factor that is expressed in early human embryonic development but subsequently silenced in somatic tissues. Importantly, DUX4, which is a key early embryonic genome activation factor, can transcriptionally upregulate *LEUTX* expression (Gawriyski et al., 2023). Additionally, WT LEUTX has been shown to physically interact with and depend on p300/CBP mediated acetylation for target gene activation (Gawriyski et al., 2023). Notably, LEUTX interacts with p300 through a 9 amino-acid transactivating domain in its C-terminus, which is conserved in the context of the CIC::LEUTX fusion (Sievers et al., 2023). Thus, we suspect that CIC::LEUTX fusion oncoproteins may in part share some overlap in their molecular targets and mode of transcriptional activation with CIC::DUX4. A recent study by Sievers et al. leveraged DNA methylation patterns to differentiate tumors harboring CIC::LEUTX fusions from other CIC-rearranged (non-CIC::LEUTX) central nervous system (CNS) sarcomas (Sievers et al., 2023). Their analysis revealed that CIC::LEUTX bearing CNS tumors segregated into its own group characterized by a pediatric-type high-grade neuroepithelial histology that was distinct from the CIC-rearranged CNS sarcoma group. Consistent with the comparison between CIC::DUX4 and CIC::NUTM1 tumors, these findings suggest that while CIC::LEUTX fusions may share some similarities with CIC::DUX4 tumors, there are likely key distinctions that differentiate between these molecular subsets. Further studies are clearly warranted to further elucidate both shared and divergent mechanisms between CIC fusions.

## Preclinical models of CIC-rearranged sarcoma

### Human cell lines

The first widely validated CIC::DUX4 patient-derived cell lines were established in 2017 by Tadashi Kondo and colleagues (Oyama et al., 2017). Specifically, a CIC::DUX4 fusion positive soft tissue tumor was derived from a 29 year-old female patient and was implanted into immunodeficient mice that was serially propagated three times to generate NCC-CDS1-X1 (cell line generated after first passage) and NCC-CDS1-X3 (cell line generated after third passage). Sanger sequencing of the explanted tumor identified an in-frame CIC::DUX4 transcript fusing *CIC* exon 20 to exon 1 of *DUX4* with a junction nucleotide sequence 5′-GGGTGGAG-3′ (Oyama et al., 2017). Eventually, Kondo and colleagues established NCC-CDS2-C1 patient-derived cell lines using a surgically resected tumor tissue from another patient with CIC::DUX4 sarcoma (CDS) containing a similar CIC::DUX4 fusion transcript (Yoshimatsu et al., 2020). Furthermore, a third cell line named Kitra-SRS was generated from a soft tissue tumor derived from a 9 year-old girl with metastatic CDS. The Kitra-SRS cell line was generated through xenograft propagation following the excision of the primary tumor. Sequence profiling revealed a fusion between *CIC* exon 20 and *DUX4* exon 1 with the 5′-GGGTGG-3′ nucleotide sequence identified at the junction between *CIC* and *DUX4*, similar to the NCC-CDS1-X1 and NCC-CDS1-X3 cells (Nakai et al., 2019). Notably, all these aforementioned models were histologically similar to the original resected CDS tumors, and Kitra-SRS tumor-bearing mice exhibited lung metastases, which recapitulated the aggressive disease pattern observed in the host from which it was derived.

While case reports identifying CIC::NUTM1, CIC::FOXO4, and CIC::LEUTX fusions in patient tumors have been increasing, there has yet to be a patient-derived cell line or xenograft generated for experimental use. We eagerly await these tools that will greatly increase our understanding of *CIC*-rearranged biology and oncogenesis.

### Murine models

Recently, Hendrickson et al. attempted to generate a transgenic CIC::DUX4 mouse model (Hendrickson et al., 2024). They engineered three CIC::DUX4 chimeric mouse models (Ch7CDS, Ai9CDS, and TOPCDS), which developed spontaneous CDS-like tumors associated with presumed widespread metastases in the lung and liver in the absence of Cre-recombinase. The authors speculate that the CIC::DUX4 fusion is a highly potent oncogene (similar to Kras) that leads to spontaneous tumor formation. These studies largely represent proof-of-principle experiments since all three chimeric models died prior to reproduction due to rapid spontaneous tumor formation and subsequent death. Despite these limitations, the authors were able to generate important molecular datasets and investigated underlying mechanisms of CIC::DUX4 mediated gene activation in mice. Collectively, their findings align with human CDS as they demonstrate multiple shared transcriptional targets including known *PEA3* family members that were, in part, associated with p300 transcriptional activation. One interesting observation that is consistent with other findings is that CIC::DUX4 localizes and binds to GGAA-motif sequences on DNA (Kim et al., 2022; Bakaric et al., 2024). These findings indicate that WT CIC and CIC::DUX4 (among other *CIC* rearrangements) may regulate gene expression through non-consensus (non TGAATGAA-like) like motifs (Thomas et al., 2023). Future studies aimed at identifying and differentiating which genes are regulated by CIC::DUX4 at TGAATGAA versus GGAA-like DNA-motifs is highly warranted.

Through CIC::DUX4 transduction of mouse embryonic mesenchymal cells (eMC), Yoshimoto et al. generated an *ex vivo* CDS mouse model that closely recapitulates the histological and clinical features of human CDS (Yoshimoto et al., 2017). In this model, CIC::DUX4 expressing eMCs were transplanted into the subcutaneous soft tissue of immunodeficient nude mice. Recipient mice rapidly developed primary tumors at 100% penetrance with a mean latency period of 24 days. Serial transplantation of these CIC::DUX4 tumors resulted in spontaneous lung metastases in 28% of recipient mice. Correlative studies further identified conserved human CIC::DUX4 transcriptional targets including *ETV1, ETV4*, and *CCND2*. The authors subsequently leveraged this mouse model to test treatment strategies and noted tumor growth inhibition using genetic and pharmacologic approaches that block the CCND2-CDK4/6 complex.

## Clinical and pathologic characteristics of CIC fusions

**FIGURE 2:**
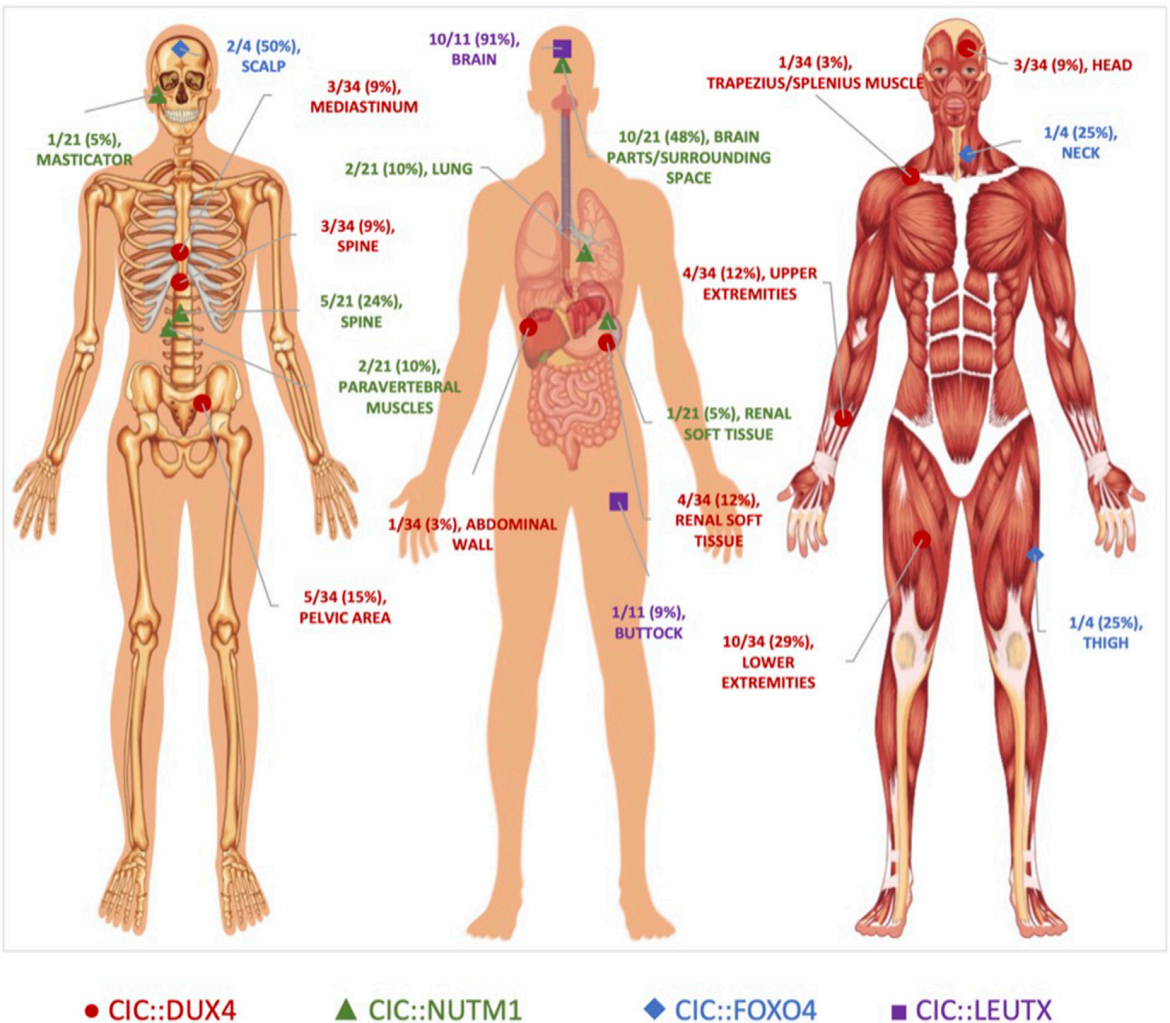
Anatomical Localization of *CIC*-rearranged Primary Tumors. Primary tumor location of CIC-fusion positive cases reported in the literature. CIC::DUX4 (red circles) tumors localized primarily in the soft tissue. CIC::NUTM1 (green triangles) tumors were predominantly observed in the central nervous system. CIC::LEUTX (purple squares) tumors were confined mostly in the brain, while CIC::FOXO4 (blue diamonds) tumors were interspersed in the scalp, neck, and thigh. Original unlabeled anatomical image from Vecton/Shutterstock.com.

In our series of CIC::DUX4 confirmed case reports median age of CDS diagnosis was 29.5 years old with a range from 11 to 82 years old and a male-to-female ratio of fourteen to eighteen (Machado et al., 2013; Bielle et al., 2014; Kajtár et al., 2014; Haidar et al., 2015; Tardío et al., 2015; Chebib and Jo, 2016; Bergerat et al., 2017; Krskova et al., 2017; Loke et al., 2017; Tsukamoto et al., 2017; Camille et al., 2018; Donahue et al., 2018; Mangray et al., 2018; Tang and Dodd, 2018; Lehane et al., 2019; Maloney et al., 2020; Ricker et al., 2020; Tamada et al., 2020; Yamada et al., 2020; Chen et al., 2021; Maejima et al., 2021; Maekawa et al., 2021; Vieira et al., 2021; Wu and He, 2022; Aranza et al., 2023; Ascione et al., 2023; Wu et al., 2023) (Table 1). Within the thirty-two CDS patients that we analyzed through case studies, ten tumors localized primarily in the soft tissue of lower extremities, with a significant fraction also presenting in the soft tissue of the upper extremities and kidney as well as pelvic cavity area. (Figure 2). In comparison, the median age of CIC::NUTM1 sarcomas is 15.5 years old (range 2–61 years old) and presents with an eight-to-six ratio of male to female incidence from the available clinical data (Table 1). Interestingly, a significant percentage of CIC::NUTM1 tumors localized to the CNS, with over 70% confined to the spine (cervical and thoracic levels) and brain (temporal and occipital areas of meninges, parenchyma, and sarcoma, trigone of the lateral ventricle, interventricular foramen, parietal lobes) with others infrequently observed in the lung and kidney soft tissue (Sturm et al., 2016; Mangray et al., 2018; Schaefer et al., 2018; Watson et al., 2018; Le Loarer et al., 2019; Biederman et al., 2022; Yang et al., 2022; Ma et al., 2023; Sievers et al., 2023) (Figure 2). The CNS tropism is consistent with prior reports (Watson et al., 2018). Thus far, only four patient case reports have been published on the CIC::FOXO4 fusion, with the median age of diagnosis of 36.5 years old (range 13–63 years old), and a three-to-one male-to-female ratio (Solomon et al., 2014; Sugita et al., 2014; Connolly et al., 2022; Babkoff et al., 2023) (Table 1). In these patients, primary tumors localized to the scalp, neck, and thigh (Figure 2).

**TABLE 1:** Clinico-pathologic characterization of CIC fusions.

Clinico-pathologic Characteristics^a^	CIC::DUX4	Antonescu et al. (2017) CIC::DUX4 data	Murphy et al. (2024) CIC::DUX4 data	CIC::NUTM1	CIC::FOXO4	CIC::LEUTX
Age (years)						
Median	29.5	31	28	15.5	36.5	7
Range	11–82	10–81	15–67	2–61	13–63	2–19
Gender (n)						
Male	14	33	2	8	3	3
Female	18	24	3	6	1	6
Metastatic rate	22/29 (76%)	30/57 (53%)	2/5 (20%)	3/12 (25%)	3/4 (75%)	Not available
Died of disease	15/24 (63%)	23/57 (40%)	2/5 (20%)	8/11 (73%)	2/4 (50%)	Not available

^a^ Based on available clinical data. Clinico-pathologic data for CIC fusions with a defined binding partner were identified through PubMed, utilizing keywords of: (“CIC-DUX4, case report”), (“CIC-NUTM1, case report”), (“CIC-FOXO4, case report”), and (“CIC-LEUTX, case report”). In addition to the individual case reports, two published studies that specifically identified CIC::DUX4 fusions in patients and provided clinical data were included.

While much less is known about the clinical features associated with CIC::LEUTX sarcoma, a recent study that profiled pediatric CNS tumors identified 9 patients with CIC::LEUTX fusions that all occurred within the supratentorial compartment (Sievers et al., 2023). The median age of onset is 7 years old (range 2–19) with a female predominance (three-to-six ratio of male to female) (Table 1). Clinical outcomes associated with CIC::LEUTX patients were available for a subset of patients (*n* = 6). Median progression-free survival was 13.5 months (range 6–16 months) with all patients developing relapse. Only one of the patients died of the disease during the follow-up period at 15 months. Treatment response was not available in this study. Another study identified two additional patients with CIC::LEUTX fusions and one was located in the midbrain/thalamus while the other was in the left buttock and associated with pulmonary metastases (Linos et al., 2023). Treatment response to radiation and temozolomide was noted in the patient with midbrain involvement, while chemotherapy (AIM) response was noted in the pulmonary metastases of the patient with the left buttock CIC::LEUTX sarcoma. Long-term clinical follow up and low numbers make it difficult to generalize these observations and more cases are needed to draw reliable conclusions about treatment response and/or anatomical distribution.

Of the four *CIC*-rearranged sarcomas, summary statistics of the available data within the case reports indicate CIC::DUX4 sarcoma to be associated with a relatively more aggressive clinical course, presenting with the highest rate of metastasis (76%; total *n* = 29) and a predilection for the lungs (Machado et al., 2013; Bielle et al., 2014; Kajtár et al., 2014; Haidar et al., 2015; Tardío et al., 2015; Chebib and Jo, 2016; Bergerat et al., 2017; Krskova et al., 2017; Loke et al., 2017; Tsukamoto et al., 2017; Camille et al., 2018; Donahue et al., 2018; Mangray et al., 2018; Tang and Dodd, 2018; Lehane et al., 2019; Maloney et al., 2020; Ricker et al., 2020; Tamada et al., 2020; Yamada et al., 2020; Chen et al., 2021; Maejima et al., 2021; Maekawa et al., 2021; Vieira et al., 2021; Wu and He, 2022; Aranza et al., 2023; Ascione et al., 2023; Wu et al., 2023). Sarcoma patients harboring the CIC::FOXO4 fusion also had a high rate of pulmonary metastasis (75%; total *n* = 4), while patients with the CIC::NUTM1 tumors had the lowest overall rate of metastasis (25%; total *n* = 12) with distant sites including the lungs, thyroid, bone, and brain (Solomon et al., 2014; Sugita et al., 2014; Mangray et al., 2018; Schaefer et al., 2018; Le Loarer et al., 2019; Biederman et al., 2022; Connolly et al., 2022; Yang et al., 2022; Babkoff et al., 2023; Ma et al., 2023; Sievers et al., 2023). Despite differences in metastatic pattern, at least half of the patients in each *CIC* rearrangement cohort, excluding CIC::LEUTX, died of their disease (Table 1).

## Current and emerging strategies to treat advanced human *CIC*-rearranged sarcomas

There is no consensus treatment strategy for patients with *CIC*-rearranged sarcomas. As a result, *CIC*-rearranged sarcomas are treated with a similar paradigm to other more chemotherapy sensitive small round cell sarcomas including Ewing sarcoma (ES). The efficacy of these conventional systemic approaches along with clinical outcomes data of patients with *CIC*-rearranged tumors who undergo multimodal treatment strategies (surgery, radiation, and chemotherapy) have been extensively discussed elsewhere (Antonescu et al., 2017; Connolly et al., 2022; Brahmi et al., 2023; Murphy et al., 2024). Therefore, in this review we will only briefly mention the standard chemotherapy backbones and largely focus on the emerging mechanism-based approaches to target *CIC*-fused sarcomas. A short list of standard chemotherapy approaches include vincristine, doxorubicin, cyclophosphamide alternating with ifosfamide, and etoposide (VDC/IE) or doxorubicin plus ifosfamide with MESNA (AIM) (Table 2). It is important to emphasize that these treatment strategies were largely based on the histological similarities between CIC::DUX4 and ES, rather than the degree of tumor sensitivity to these chemotherapy regimens (Connolly et al., 2022).

**TABLE 2:** Attempted and proposed treatment modalities for *CIC*-rearranged sarcomas.

*CIC*-rearranged sarcoma	Attempted chemotherapeutic approaches	Proposed targeted therapy mechanisms
CIC::DUX4	VDC/IE AIM	Dinaciclib (CDK2 inhibitor)
		Adavosertib (WEE1 inhibitor)
		Linsitinib (IGF-1 receptor inhibitor) iP300w (p300/CBP inhibitor)
		Anti-DUX4 antibodies
		BCI (DUSP6 inhibitor)^a^
CIC::NUTM1	IVA	
	AI	
	Etoposide/carboplatin/IVADo	
	VIDE	
	BEP	
CIC::FOXO4	VDC/IE	Cabozantinib (receptor tyrosine kinase inhibitor)
CIC::LEUTX		

Summary of chemotherapy backbones that have been used for each respective CIC fusion is noted in Table 2. Mechanism-based targeted therapies have identified potential dependencies predominantly in CIC::DUX4 sarcoma. Few therapies exist beyond conventional chemotherapy for CIC::NUTM1 and CIC::LEUTX fusions, with one targeted therapy attempted in a patient with CIC::FOXO4.

•  VDC/IE: vincristine, doxorubicin, cyclophosphamide alternating with ifosfamide, and etoposide.

•  AIM: doxorubicin, ifosfamide, MESNA.

•  IVA+/−Do: ifosfamide, vincristine, dactinomycin, +/− doxorubicin.

•  VIDE: vincristine, ifosfamide, doxorubicin, etoposide.

•  BEP: bleomycin, etoposide, cisplatin.

^a^ Not in clinical use.

Of the four CIC::FOXO4 sarcoma patients featured in the literature, two died of their disease despite utilization of surgical resection, adjuvant chemotherapy composed of common therapeutic combinations (e.g., VDC/IE), or use of kinase inhibitors (e.g., cabozantinib on clinical trial) (Solomon et al., 2014; Sugita et al., 2014; Connolly et al., 2022; Babkoff et al., 2023) (Table 2). Although CIC::NUTM1 has the lowest metastatic rate amongst the *CIC*-rearranged sarcomas, eight of the fourteen patients featured in the case reports died of their disease despite only two experiencing metastatic progression and amidst the use of surgical resection, chemotherapy, and radiotherapy in most of these patients (Le Loarer et al., 2019; Yang et al., 2022). One of the patients specifically followed the ES chemotherapeutic combination regimen of six cycles of VIDE (vincristine, ifosfamide, doxorubicin, and etoposide) but unfortunately continued to progress. Functional studies to elucidate the molecular pathogenesis of CIC::NUTM1 and CIC::FOXO4 sarcomas and identify therapeutic targets are warranted.

### Mechanism-based targeted therapies in *CIC*-rearranged sarcomas

Although the CIC::DUX4 fusion was the first *CIC*-rearranged sarcoma identified in 2006, there are still inadequate chemotherapy regimens utilized for CDS. Recent studies have focused on understanding the mechanistic underpinnings of the CIC::DUX4 fusion oncoprotein, illuminating potential druggable targets (Table 2). Preclinical studies identified CIC::DUX4 to molecularly depend on cell cycle mediators CCNE1 and WEE1 (Okimoto et al., 2019; Ponce et al., 2022) with *CCNE1* being established as a direct transcriptional target of CIC::DUX4 that drives tumor growth and survival through an acquired dependence on CCNE/CDK2 complex. Hyperactivation of the CCNE/CDK2 complex compromises the G1/S transition and confers sensitivity to CDK2 inhibitors in a relatively fusion specific manner (Okimoto et al., 2019). In order to prevent the accumulation of DNA damage in S phase and to subsequently prevent premature mitotic entry, CIC::DUX4 sarcomas depend on the G2/M checkpoint kinase WEE1 to delay mitotic entry and to ensure proper DNA repair and integrity prior to mitosis. Thus, WEE1 kinase inhibition with adavosertib has been shown to induce tumor regression in CIC::DUX4 tumor xenograft models (Ponce et al., 2022). These preclinical studies provide rationale to develop CDK2 and WEE1 inhibitor–based clinical trials for CIC::DUX4 patients. To this end, Blueprint Medicine is testing their CDK2 inhibitor (BLU-222) in patients with *CIC*-rearranged tumors through their ongoing clinical trial, NCT05252416 (Table 2). Additionally, based on the preclinical findings mentioned above, we and others are working to develop a future WEE1-directed strategy to test in patients with *CIC*-rearranged tumors.

Another mechanism-based approach to target CDS was identified through the development and study of the Kitra-SRS CIC::DUX4 positive cell line (Nakai et al., 2019), which was discussed above. Specifically, analysis of Kitra-SRS cells revealed autocrine activation of the insulin-like growth factor 1 (IGF-1)/IGF-1 receptor (IGF-1R) signaling pathway (Table 2). Thus, inhibiting this pathway with the IGF-1R inhibitor, linsitinib, limited IGF-1R/AKT signaling and reduced Kitra-SRS tumor growth both *in vitro* and *in vivo*.

Since MAPK-ERK signaling leads to WT CIC degradation, Lin et al. hypothesized that pharmacologic activation of ERK could potentially lead to direct degradation of the CIC::DUX4 oncoprotein, which retains the highly conserved ERK binding domain. Through a series of biochemical and genetic studies, they demonstrated that ERK activation through inhibition of the DUSP6 phosphatase (dephosphorylates ERK to decrease activity) could lead to CIC::DUX4 degradation. Moreover, they identify that CIC::DUX4 transcriptionally upregulates DUSP6 to silence ERK activity and sustain CIC::DUX4 oncoprotein expression in sarcoma (Lin et al., 2020). The use of DUSP6 inhibitors is not in clinical use largely due to the potential negative effects of activating ERK in non-CIC::DUX4 sarcoma (Table 2).

A broader approach to target CIC::DUX4 transcriptional activity was identified by Bosnakovski and colleagues. Leveraging biological insight of how WT DUX4 (and CIC::DUX4) interacts with p300, they rationalized that p300 inhibition could potentially overcome CIC::DUX4 sarcoma growth and survival. They demonstrated that a iP300w, a potent p300/CBP inhibitor, reversed CIC::DUX4 associated histone H3 acetylation marks and consequently suppressed CIC::DUX4 transcriptional activity while limiting tumor growth (Bosnakovski et al., 2021) (Table 2).

Finally, therapies that directly target components of the CIC::DUX4 fusion oncoprotein are emerging. Specifically, Targeted RNAi Molecules (TRiM) are being employed to intercept DUX4 in facioscapulohumeral muscular dystrophy (NCT06131983). Cell penetrating antibodies designed to target intracellular neoantigens including fusion oncoproteins are also in early development and could be of clinical interest in the near future (Herrmann et al., 2019). Anti-DUX4 strategies are of potential interest largely since WT DUX4 is typically silenced in adult somatic tissues (Karpukhina et al., 2021). Thus, RNAi-based technologies and/or cell penetrating antibodies that can effectively target the DUX4 in the context of the CIC::DUX4 fusion can open another therapeutic avenue directed at selective targeting of the CIC::DUX4 oncoprotein in human sarcoma (Table 2).

Collectively, we hope that future and ongoing studies aimed at identifying and exploiting key dependencies in CIC::DUX4 sarcoma will apply to other *CIC*-rearranged sarcomas to improve outcomes for these patients who currently have no therapeutic options.

## Discussion

CIC::DUX4 was first recognized in a subset of undifferentiated small round cell sarcomas that lacked conventional EWSR1 fusions that characterize ES. Improved model systems coupled with more advanced profiling studies have enabled a deeper understanding into the biological underpinnings of CIC::DUX4 mediated oncogenesis. Moreover, enhanced sequencing technologies and pathological stratification have led to the discovery of more CIC-rearranged proteins including CIC::NUTM1, CIC::FOXO4, and CIC::LEUTX. On the structural level, these fusions share a significant fraction of WT CIC including its DNA-binding domains and target gene specificity. Early data suggest that the developmentally regulated C-terminal binding partners (DUX4, NUTM1, FOXO4, and LEUTX) may confer activating capacity through the recruitment of the p300/CBP complex. However, further studies are needed to validate this hypothesis. Despite their commonalities in fusion structure, we anticipate transcriptional and perhaps functional divergence between CIC fusion types, which is supported through the aforementioned transcriptional studies comparing CIC::DUX4 to CIC::NUTM1 and/or CIC::LEUTX tumors which are closely but not identically clustered. These subtle molecular differences may provide insight into the key differences that differentiate *CIC* rearrangements in patients, including anatomical tropism, metastatic potential, and response to therapy.

Development of preclinical cell-based and animal models to further dissect the molecular and functional mechanisms of not only CIC::DUX4 but also the newly recognized CIC::NUTM1, CIC::FOXO4, and CIC::LEUTX chimeras will provide insight to improve treatment strategies for these patients. We applaud rare tumor programs that aim to develop new patient-derived models, such as *CIC*-rearranged sarcoma cell lines that can be subsequently shared with the scientific community to improve our understanding through mechanistic studies.

Due to its rarity, the development of new therapeutic strategies to target CIC::DUX4 sarcoma remains limited. Through deep mechanistic dissection of CIC::DUX4 biology we and others have identified preclinical targets that warrant clinical validation to refine the treatment paradigm for patients with *CIC*-rearranged sarcoma. We encourage clinical investigators to work together on a national or international level to enroll patients with *CIC*-rearranged sarcomas on ongoing and/or future mechanism-based clinical trials.

Another important barrier to overcome is within the clinical realm, calling for the unified differentiation by the sarcoma community to distinguish *CIC*-rearranged sarcomas from other small round cell sarcomas such as ES (Blay et al., 2016). This is of particular importance given the overall lack of therapeutic consensus and misinformed treatment strategies that patients with CIC fusions often face in the clinic. One strategy to overcome the barriers of enrolling patients with rare *CIC*-rearranged sarcomas onto clinical trials is to leverage the National Cancer Institute (NCI)-sponsored Experimental Therapeutics Clinical Trials Network (ETCTN). ETCTN participating sites throughout the United States can collaborate to conduct early trials that target *CIC*-rearranged sarcoma with NCI-IND agents, such as WEE1 inhibitors. Collaboration between the scientific and clinical community is essential in order to combat these rare but lethal subtypes of sarcoma with novel and effective molecular-based therapeutic interventions.
